# Risk of pediatric obstructive sleep apnea in preadolescent children with orthodontic anomalies: a diagnostic study

**DOI:** 10.3389/froh.2026.1868634

**Published:** 2026-08-25

**Authors:** Zuzana Marincak Vrankova, Marek Vranka, Jan Bohm, Pavel Hornik, Petra Borilova Linhartova

**Affiliations:** 1 Arculum – Medical Center, Brno, Czech Republic; 2 Clinic of Stomatology, St. Annes University Hospital, Brno, Czech Republic; 3 Clinic of Maxillofacial Surgery, University Hospital Brno, Brno, Czech Republic; 4 RECETOX, Faculty of Science, Masaryk University, Brno, Czech Republic; 5 Faculty of Social Sciences, Charles University, Prague, Czech Republic; 6 Children ENT Ltd., Brno, Czech Republic

**Keywords:** ANB angle, apnea-hypopnea index, crowding, maxillary arch constriction, oral breathing, orthodontic malocclusions, polygraphy

## Abstract

**Background:**

This study aimed to investigate the relationships among the apnea-hypopnea index (AHI) measured by home sleep apnea testing (HSAT) as a screening indicator of the likelihood of pediatric obstructive sleep apnea (POSA), the presence of orthodontic malocclusions, and oral breathing.

**Methods:**

Children aged 6 to 12 years, of (nationality) or (nationality) nationality, who were referred for orthodontic examination were included in the study. Evaluated cranio-maxillofacial features included maxillary arch constriction, anomalies in the number and position of teeth, dental arch relationship, skeletal class, mandibular growth pattern, and tongue range of motion ratio. The likelihood of POSA was screened using overnight home respiratory polygraphy (HSAT), with the apnea-hypopnea index (AHI) as the quantitative primary outcome; HSAT is a screening tool and does not replace full-night polysomnography (PSG), the diagnostic gold standard. A stepwise linear regression model was developed to evaluate the relationship between clinically significant variables and apnea-hypopnea index (AHI).

**Results:**

A total of 100 children participated in the study, with 61% having an AHI consistent with mild POSA and 7% with moderate POSA on HSAT screening, respectively. AHI (treated as a continuous variable) positively correlated with maxillary arch constriction, overjet, increased ANB angle, mandibular crowding, and oral breathing preference, and negatively with the tongue range of motion ratio (correlation coefficients 0.2–0.47). The final linear regression model retaining only four parameters (maxillary arch constriction, mandibular crowding, increased ANB angle, and oral breathing preference) yielded AUC of 0.72 and 0.91 for the identification of children at risk of POSA and moderate POSA, respectively.

**Conclusions:**

Our study confirmed the multifactorial nature of POSA and the elevated risk of POSA in the orthodontic population. Including craniofacial features with small effect sizes in the model improved its performance, highlighting the importance of considering multiple contributing factors rather than isolated predictors when assessing POSA risk.

## Introduction

Sleep disorders are relatively common in the population, affecting not only adults but also children and adolescents. According to a recent population-based study, the prevalence of sleep disorders in young adult patients may be as high as 42.3% (1). In the pediatric population, the disruption of the quality and quantity of sleep carries also risks related to their growth and overall development (2). Pediatric obstructive sleep apnea (POSA), one of sleep-related breathing disorders (SRBD), is a multifactorial disease with complex pathogenesis including both genetic and environmental factors (3). The prevalence of POSA varies significantly, generally estimated to be between 1% and 5%, although the systematic review by Magnusdottir and Hill reported higher prevalence ranging from 12.8% to 20.4% in recent years (2016–2023). This may be caused by the differences in diagnostic criteria and methods used (4, 5) or specifics of studied population. The pediatric orthodontic population represents specific group who are at higher risk of SRBD, including POSA (6).

POSA most commonly affects children between two and eight years of age, usually in association with significant growth of the lymphoid tissue within the upper respiratory tract during this period (3, 7). The most common symptoms and complications of POSA include daytime fatigue, drowsiness or, vice versa, hyperactivity, inability to concentrate, and problems at school. Even behavioral problems or personality changes may occur. If a severe form of POSA is left untreated for a long time, the symptoms may include hypertension, acute heart attack, and/or cardiopulmonary failure (7–10).

With the growing awareness of this issue, the emphasis put on early diagnosis and therapy initiation is also increasing. However, despite this knowledge, no study on the prevalence of POSA in the (Central Europe) has been published so far. Based on the literature and clinical experience, we hypothesized that the presence of specific craniofacial features predisposes the child to a higher risk of POSA, and, therefore, that the prevalence of POSA in these children may increase in comparison with the prevalence reported in the general population (11, 12). It is also recognized that upper airway obstruction due to enlarged adenoids and tonsils can contribute to the development of specific craniofacial features, highlighting the complex interplay between airway obstruction and craniofacial development (13).

In this cross-sectional observational study, we have (i) focused specifically on the prevalence of the high risk of POSA (as determined by home sleep apnea test, HSAT) in children referred for an orthodontic examination. In addition, (ii) we also aimed to investigate the associations between the risk of POSA characterized by the apnea-hypopnea index (AHI) and craniofacial anomalies as well as oral breathing in these patients, (iii) to build a linear regression model based on clinically relevant craniofacial features correlating with AHI in pediatric orthodontic patients, and (iv) to highlight the need for interdisciplinary cooperation in the diagnosis and management of sleep disorders.

## Materials and methods

**Figure 1:**
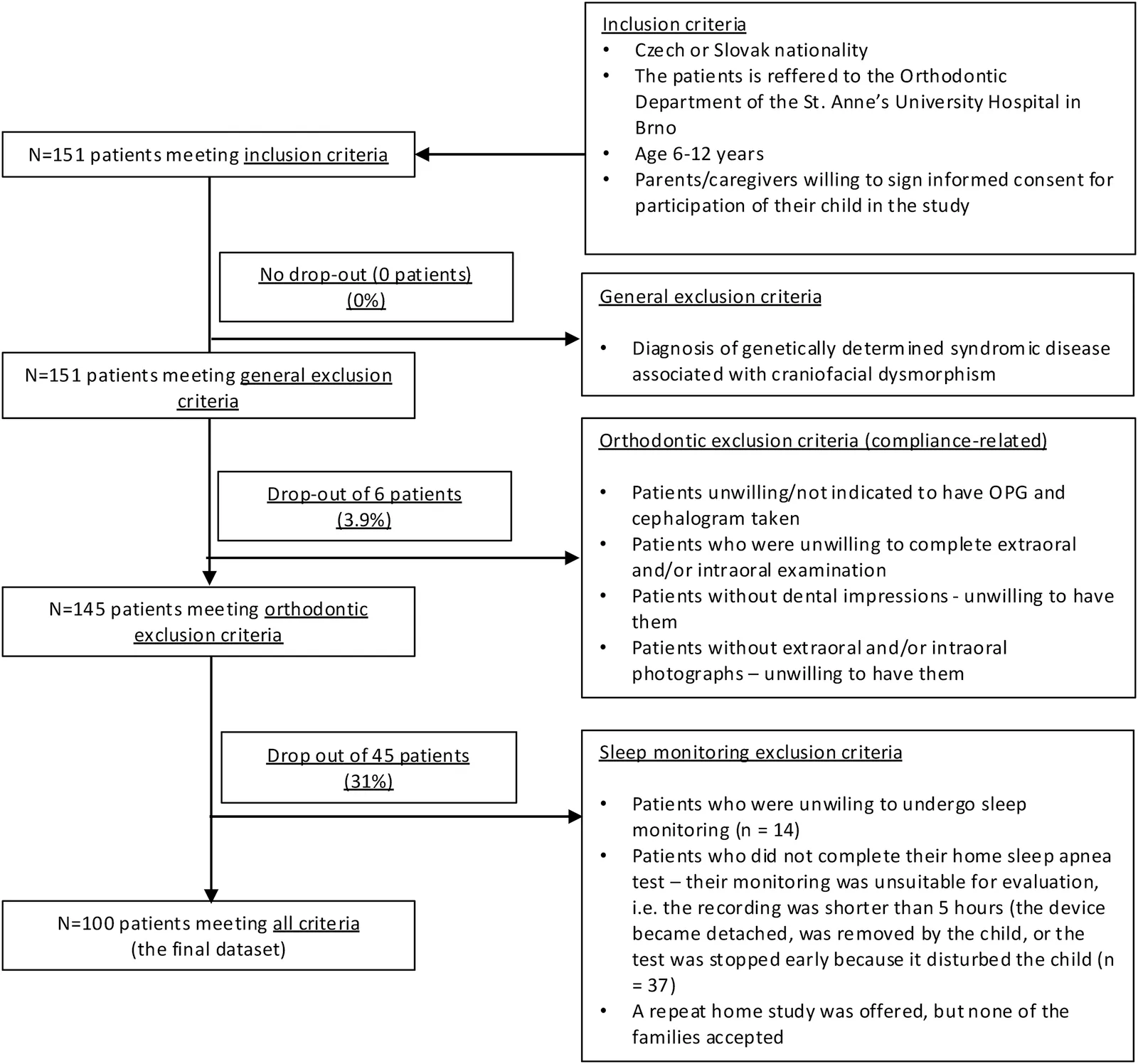
Flowchart of inclusion and exclusion criteria for the study. The diagram shows the 151 initially eligible patients, the 6 patients excluded at the orthodontic stage due to non-compliance with orthodontic examination, further drop-out at the sleep-monitoring stage (39 due to refusal to undergo testing and 6 due to the inadequate recording), yielding the final sample of 100 patients; a repeat measurement was offered to families submitting inadequate recording but none accepted.

This single-center, cross-sectional observational diagnostic study was approved by the St. Anne's University Hospital Ethics Committee. Craniofacial and orthodontic characteristics and sleep-related parameters were assessed within the same study period for each participant, with no longitudinal follow-up. In line with the Helsinki Declaration, informed consent was obtained from all parents/caregivers (hereinafter referred to as parents) of every child prior to the inclusion in the study. Children who were referred to the Orthodontic Department of the Clinic of Stomatology, St. Anne's University Hospital, Brno, Czech Republic and met the following inclusion criteria were approached to participate in this study prior to the start of their orthodontic treatment: a) age 6 to 12 years, b) (Czech) or (Slovak) nationality to maintain the homogeneity in the sample and control for genetic and environmental factors, and c) parents/caregivers willing to sign consent for participation of their child in the study. A diagnosis of a genetically determined syndromic disease associated with craniofacial dysmorphisms, such as Down syndrome, Pierre Robin sequence, Apert syndrome, Crouzon syndrome, etc., was an exclusion criterion (see the Figure 1 to illustrate patient recruitment, participation, and exclusion details).

### Patient recruitment

The flowchart in Figure 1 details the patient selection and exclusion process for our study.

All home recordings were manually reviewed and scored in Sleepware G3 (v3.9.6.0) by a doctor trained in sleep medicine, who removed obvious non-sleep periods and assessed signal quality; a recording was deemed non-interpretable when fewer than 5 h of analyzable, artifact-free signal were available to derive a reliable AHI.

Ultimately, the final dataset comprised 100 patients who met all the study requirements and exclusion criteria.

### Clinical examinations

A comprehensive patient history was obtained during the initial examinations (the same doctor in all cases). Body Mass Index (BMI) and corresponding z-scores for age and sex were calculated using the Epi Info TM software (Centers for Disease Control and Prevention, CDC; in Atlanta, Georgia, USA), following WHO guidelines 2007 for children and adolescents (5–19 years) (14). BMI classification was based on the patients’ z-scores. Each patient underwent HSAT using the Alice NightOne device (Respironics, Inc., Murrysville, PA 15668, USA). HSAT (home respiratory polygraphy) is a validated screening modality for sleep-disordered breathing; however, full-night polysomnography (PSG) remains the diagnostic gold standard for POSA. Accordingly, all sleep-related findings in this study, including the reported prevalence and severity categories, represent screening-based estimates of the likelihood of POSA derived from the AHI rather than PSG-confirmed diagnoses. For brevity, the terms “AHI consistent with (mild/moderate) POSA on screening” are used throughout this manuscript to denote the screening-level likelihood of POSA rather than a confirmed diagnosis.

The device recorded the AASM-recommended channels for HSAT: body position, airflow (nasal pressure), snoring, respiratory effort, oxygen saturation (SpO_2_), and pulse. Respiratory events were scored manually according to the American Academy of Sleep Medicine (AASM) Manual for the Scoring of Sleep and Associated Events (version 2.6, 2020) (15), with apnea defined as a drop in the signal excursion by ≥90% of the pre-event baseline for ≥10 s or two breath cycles, and hypopnea was defined as a drop by ≥30% of the pre-event baseline using nasal pressure, followed by a ≥ 3% oxygen desaturation All recordings were manually scored in Sleepware G3 (v3.9.6.0; Respironics, Inc., Murrysville, PA, USA) by a doctor trained in sleep medicine, and selected records were additionally consulted with the Cardiovascular Sleep Centre. Measurements were considered interpretable only when a minimum of 5 h of artifact-free, analyzable recording with adequate signal quality on the airflow and SpO_2_ channels was obtained (16, 17); recordings not meeting this quality-control threshold were classified as insufficient for evaluation. The pediatric AHI severity cut-offs and the full acquisition and scoring protocol are detailed in the Supplementary Methods. Because home respiratory polygraphy lacks EEG, the recorded index is conventionally a respiratory event index (REI) referenced to the total recorded time (TRT), which can underestimate disease severity. To approximate the total sleep time (TST) and thereby report an apnea-hypopnea index (AHI) rather than an REI, the sleep-trained doctor manually reviewed each record and removed obvious non-sleep periods (see the Supplementary Methods).

An orthodontic examination included extraoral and intraoral assessments, photographs, dental impressions, and x-rays. Tongue mobility was assessed using the tongue range of motion ratio (TRMR) according to the method by Marchesan (18). With a stainless-steel tongue-tie assessment tool, we measured the maximal interincisal opening (MIO) and the interincisal opening with the tongue tip held against the maxillary incisive papilla (MOTTIP), and calculated TRMR = (MOTTIP/MIO) × 100%. A TRMR > 60% was interpreted as physiological tongue mobility, 51%–59% as an unclear result, and < 50% as restricted mobility consistent with a short/abnormal sublingual frenulum. Standardized lateral cephalograms and orthopantomograms were acquired with a CS 9300 unit (Carestream Dental, USA) and analyzed using the Ortik cephalometric software (Orthosoft s.r.o., Czech Republic) with digital tracing and manual identification of cephalometric landmarks. The cephalometric variables assessed were the SNA, SNB, ANB, and SN-ML angles and the hyoid bone position (AH-AH’, the distance of the hyoid bone from the mandibular line). Reference ranges followed Jarabak and Fizzell (19): skeletal class I for ANB between −1° and 5°, class II for ANB > 5°, and class III for ANB < −1°; and a neutral (28–36°), hyperdivergent (SN-ML > 36°), or hypodivergent (SN-ML < 28°) growth pattern. Landmark definitions and the measurement-error analysis are detailed in the Supplementary Methods and Supplementary Table S1. The presence of uni- or bilateral crossbite was examined, and maxillary arch constriction was measured on digital dental models. All plaster casts were scanned with an iTero Element 5D Plus intraoral scanner (Align Technology Inc., San Jose, CA, USA), and constriction was quantified as the difference between the maxillary and mandibular intermolar widths (between the mesiopalatine cusps of the upper first molars and the fissures of the lower first molars), with maxillary arch constriction exceeding 4 mm regarded as an anomaly. As a second step, we have also combined it with the evaluation of the WALA ridge reference (the most lateral and inferior aspect of the alveolar bone on the mandible, at the level of the mucogingival junction, initially described by Andrews and Andrews) to account for discrepancy that could be masked by the inclination of the lower molars (20). On the scanned mandibular models, the distance between the WALA ridge—the most lateral and inferior aspect of the mandibular alveolar bone at the level of the mucogingival junction (20)—and the facial axis (FA) point was measured separately for the right and left first molars; deviation of this distance from the 2 mm reference described by Andrews and Andrews was used to correct the intermolar-width comparison. Because the 2 mm reference may be arbitrary in children (21), the maxillary intermolar width alone was also analyzed. All clinical, model-based, and cephalometric measurements were performed by a single calibrated examiner following standardized protocols, which minimizes interobserver variability. To quantify intraobserver reliability, each of the 100 cephalograms and each of the 100 digital model scans was measured three times in succession at 14-day intervals, with re-identification of all cephalometric landmarks at every session. Intraobserver agreement was excellent (intraclass correlation coefficient [ICC] 0.991–0.999), the relative Dahlberg error ranged from 0.3% to 10.8%, and a paired t-test revealed no systematic error for any parameter (all *p* > 0.05). The full measurement protocol and method-error analysis are provided in the Supplementary Methods and Supplementary Table S1.

ENT examinations were performed in 48 children and involved a detailed endoscopic assessment of nasal and nasopharyngeal conditions, with staging of tonsil and adenoid sizes. The statistical analysis of these features was based solely on the 48 patients who had this examination performed. The patient history, as provided by the parents, specifically included questions regarding prior surgical interventions such as adenotonsillectomy or adenotonsillotomy. However, we observed inconsistencies between the surgical histories reported by the parents and the findings from the ENT examinations. Therefore, we did not include this information in our analysis unless it was confirmed by the ENT specialist during the examination. All clinical, orthodontic, cephalometric, and ENT examination procedures are described in detail in the Supplementary Methods.

### Statistical analysis

The data in this study were analyzed using R, Version 4.1.2. For the description of the dataset, we utilized the Table 1 R package. Since most of the observed variables (with the exception of BMI z-score) failed the Shapiro–Wilk test of normality, the Kruskal–Wallis test was employed for numerical and ordinal variables, while Fisher's exact test was used for categorical variables. This approach allowed us to comprehensively summarize and compare the characteristics of the dataset. The co-occurrence of dichotomous variables was investigated using the UpSet plot from the library ComplexUpset.

**Table 1:** Descriptive statistics for measured clinical variables in participants stratified by their HSAT-based AHI severity category (a screening indicator of the likelihood of POSA, not a polysomnography-confirmed diagnosis).

Clinical variables	All patients (*N* = 100)	Patients with physiological finding (*N* = 32)	Patients with mild POSA (*N* = 61)	Patients with moderate POSA (*N* = 7)	*p*-value
**AHI** median [min; max]	1.8 [0; 8.2]	0.70 [0; 1.0]	2.2 [1.1; 4.0]	6.6 [5.1; 8.2]	<0.001
Mean (± SD)	2.0 (± 1.6)	0.60 (± 0.36)	2.3 (± 0.75)	6.6 (± 1.1)	
**Sex** (male)	44 (44%)	14 (44%)	26 (43%)	4 (57%)	0.939
**Age** median [min; max]	9.5 [7.0; 12]	10 [7.0; 12]	10 [7.0; 12]	9.0 [7.0; 10]	0.289
Mean (± SD)	9.6 (± 1.7)	9.9 (± 1.8)	9.6 (± 1.7)	8.6 (± 0.98)	
**BMI z-score** Median [min; max]	0.20 [−3.9; 3.4]	0.36 [−3.9; 1.9]	0.012 [−3.0; 3.4]	−0.18 [−2.4; 1.2]	0.904
Mean (± SD)	0.13 (± 1.3)	0.15 (± 1.4)	0.16 (± 1.3)	−0.34 (± 1.5)	
Underweight/normal/overweight/obese	7/66/22/5	2/19/11/0	3/44/9/5	2/3/2/0	
**Anterior crossbite**	3 (3%)	1 (3%)	2 (3%)	0 (0%)	1
**Posterior crossbite**	17 (17%)	4 (12%)	10 (16%)	3 (43%)	0.318
**Single tooth crossbite**	2 (2%)	1 (3%)	1 (2%)	0 (0%)	0.849
**Supernumerary tooth**	1 (1%)	1 (3%)	0 (0%)	0 (0%)	0.453
**Tooth agenesis**	7 (7%)	4 (12%)	3 (5%)	0 (0%)	0.803
**Tooth retention**	8 (8%)	5 (16%)	2 (3%)	1 (14%)	0.124
**Mandibular crowding** (mm)					
Median [min; max]	3.5 [0; 11]	3.0 [0; 6.3]	3.0 [0; 11]	5.9 [2.1; 8.0]	0.070
Mean (± SD)	3.2 (± 2.3)	2.7 (± 1.8)	3.2 (± 2.4)	5.4 (± 2.1)	
**Maxillary crowding** (mm)					
Median [min; max]	3.9 [0; 11]	3.9 [0; 8.6]	3.6 [0; 10]	5.9 [2.5; 11]	0.187
Mean (± SD)	3.8 (± 2.7)	4.0 (± 2.4)	3.4 (± 2.7)	5.8 (± 2.6)	
**Maxillary arch constriction** (mm)					
Median [min; max]	3.8 [−0.90; 12]	2.7 [0.10; 6.2]	3.8 [−0.90; 12]	7.2 [4.4; 11]	0.001
Mean (± SD)	3.8 (± 2.4)	2.9 (± 1.6)	3.9 (± 2.4)	7.1 (± 2.4)	
**Overbite** (mm)					
Median [min; max]	3.4 [−8.1; 7.4]	3.5 [−1.5; 5.9]	3.4 [−8.1; 7.4]	2.9 [−6.0; 7.1]	0.924
Mean (± SD)	2.8 (± 2.9)	3.1 (± 1.5)	2.7 (± 3.2)	1.4 (± 4.8)	
**Overjet** (mm)					
Median [min; max]	3.4 [−3.5; 12]	2.6 [−3.5; 7.9]	4.5 [−3.1; 12]	5.1 [1.8; 9.0]	0.098
Mean (± SD)	4.2 (± 2.7)	3.2 (± 2.4)	4.6 (± 2.8)	5.2 (± 2.9)	
**ANB angle** (°)					
Median [min; max]	4.3 [−2.9; 9.9]	3.2 [−2.3; 9.8]	4.5 [−2.9; 9.9]	6.8 [0.10; 9.9]	0.261
Mean (± SD)	4.0 (± 2.7)	3.5 (± 2.6)	4.0 (± 2.7)	5.7 (± 3.1)	
**SN-ML angle (°)**					
Median [min; max]	34 [18; 55]	32 [18; 41]	34 [21; 55]	36 [31; 38]	0.593
Mean (± SD)	33 (± 5.6)	33 (± 5.2)	33 (± 6.1)	35 (± 2.4)	
**AH-AH'**					
Median [min; max]	10 [3.0; 22]	10 [4.0; 18]	9.0 [3.0; 21]	14 [5.0; 22]	0.25
Mean (± SD)	10 (± 3.9)	11 (± 3.0)	9.7 (± 3.9)	13 (± 6.0)	
**TRMR** (%)					
Median [min; max]	75 [42; 100]	75 [48; 100]	77 [42; 96]	60 [42; 86]	0.148
Mean (± SD)	73 (± 15)	74 (± 15)	74 (± 14)	60 (± 14)	
**Daytime OB**	50 (50%)	8 (25%)	35 (57%)	7 (100%)	<0.001
**Nighttime OB**	70 (70%)	21 (66%)	42 (69%)	7 (100%)	0.411
Missing	1 (1.0%)	1 (3.1%)	0 (0%)	0 (0%)	
**Nighttime and daytime OB**	47 (47%)	7 (22%)	33 (54%)	7 (100%)	<0.001
Missing	1 (1.0%)	1 (3.1%)	0 (0%)	0 (0%)	
**Palatine tonsils**					
Grade 0/1/2/3/missing	2/11/33/2/52	0/7/7/0/18	2/4/23/2/30	0/0/3/0/4	0.408
**Nasopharyngeal tonsils**					
Grade 1/2/missing	41/7/52	13/1/18	25/6/30	3/0/4	0.811

AH-AH’, the distance of the hyoid bone from the mandibular line; AHI, apnea-hypopnea index; physiological finding, AHI 0–1; mild POSA, AHI 1–5; moderate POSA, AHI 5–10; ANB, angle between points A, nasion, and B; BMI, body mass index; missing, missing values; HSAT, home respiratory polygraphy; N, number of patients; nasopharyngeal tonsils grade 1, filling one-third of the vertical portion of the choanae; nasopharyngeal tonsils grade 2; one-third to two-thirds of the choanae; OB, oral breathing; palatine tonsils grade 0, tonsillectomy; palatine tonsils grade 1, hidden within the tonsil pillars; palatine tonsils grade 2, extending to the pillars; palatine tonsils grade 3, beyond the pillars; POSA, pediatric obstructive sleep apnea; SD, standard deviation; SN-ML (sellanasion/mandibular line) angle; TRMR, tongue range of motion ratio; the *p*-values were calculated using the Kruskal–Wallis test for numerical variables and the Fisher exact test for categorical variables.

To analyze the relationships between various types of variables, we employed different statistical methods as appropriate for each variable type. For numerical and ordinal variables, the Spearman correlation coefficient was utilized to assess the strength and direction of the associations. In the case of dichotomous categorical variables, we used the absolute value of the rank-biserial correlation coefficient (from the library effectsize) in conjunction with the Wilcoxon test to examine the correlations between numerical or ordinal variables and dichotomous variables. To investigate the relationship between two dichotomous variables, we applied Cramer's V (library lsr) alongside Fisher's exact test. The final correlation matrix was visualized using the corrplot package.

To better understand the contribution of individual variables to the risk of POSA, a multivariable linear regression model was constructed with AHI as the dependent variable. Candidate predictors included demographic and anthropometric variables (age, sex, and BMI z-score), and all craniofacial and functional variables that showed significant associations with AHI in the univariate analyses. Model selection was performed using bidirectional stepwise regression to identify the most parsimonious set of predictors. Multicollinearity was assessed using the generalized variance inflation factor (GVIF). The final model was validated using repeated random training-validation splits (80% training, 20% validation; 1,000 repetitions). Model performance was evaluated using the coefficient of determination (R^2^), mean squared prediction error (MSE), normalized root mean squared error (NRMSE), classification accuracy for predefined AHI severity categories, and receiver operating characteristic (ROC) analysis.

## Results

### Description of the dataset

Initially, 151 patients met the aforementioned inclusion and exclusion criteria. However, 51 of them (33.8%) did not reach the final analysis: 6 (3.9%) at the orthodontic stage and 45 (31.0% of the 145 who completed the orthodontic examination) at the sleep-monitoring stage, of which 39 due to refusal to undergo sleep monitoring and 6 due to the failure to yield evaluable home sleep apnea test (i.e., a recording containing fewer than 5 h of analyzable signal because of the device becoming detached, the child removing it, or because the recording was stopped early because it disturbed the child).

A total of 100 children were, therefore, included in the final analysis, with detailed characteristics provided in Table 1. Although our age inclusion criterion allowed the inclusion of 6–12-year-old children, no child in our study group was younger than 7 years (mean age ± standard deviation, 9.6 ± 1.7 years). BMI categorization determined based on the z-score classified 7% of children as underweight, 66% were in the normal weight range, 22% were overweight, and 5% were obese. Based on the HSAT examination, only 32 children out of 100 had a physiological finding, i.e., AHI 0–1; an AHI within the interval 1–5, consistent with mild POSA on HSAT screening, was detected in 61% of children, and 7% of the children had an AHI within the range of 5–10, consistent with moderate POSA. No child had an AHI > 10 (the category associated with severe POSA). These HSAT-based AHI categories indicate the likelihood and screening-level severity of POSA and do not constitute PSG-confirmed diagnoses (see the Supplementary Methods for the cut-offs).

**Figure 2:**
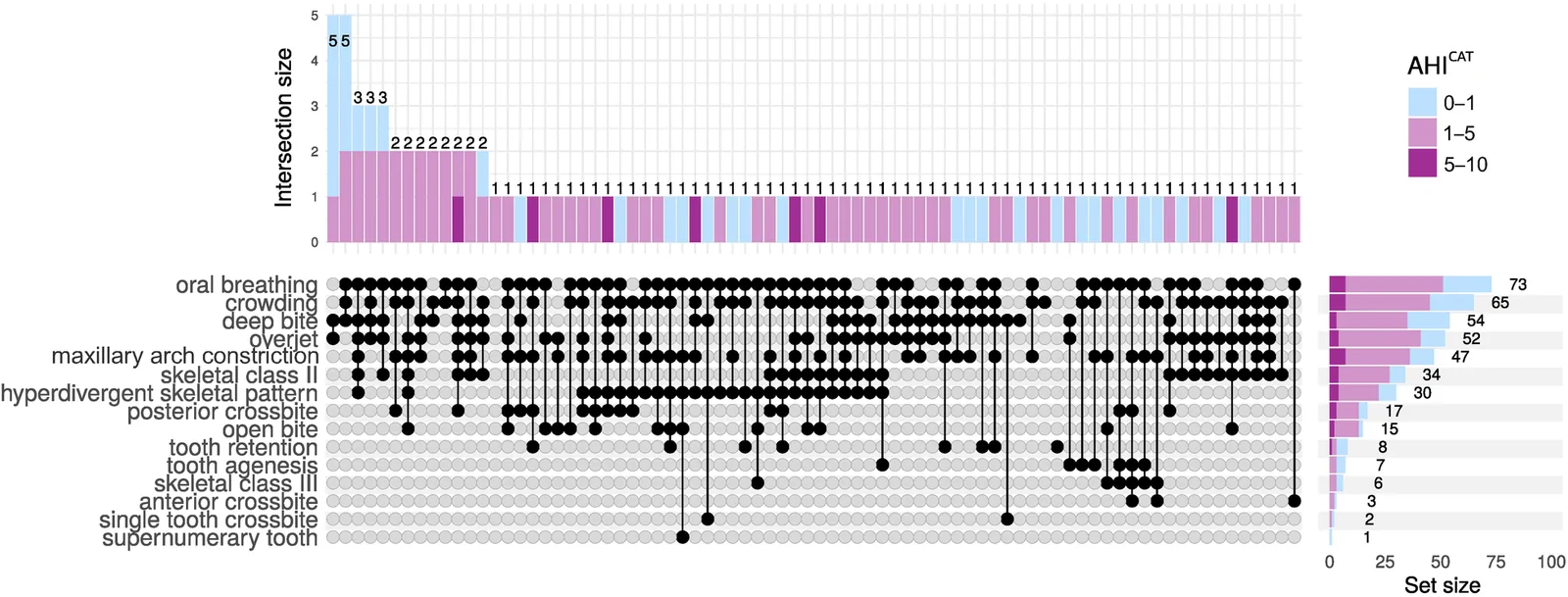
Upset plot – orthodontic malocclusions (categorically) and their co-occurrence in the study group. The nighttime oral breathing parameter is not included in the UpSet plot due to the one missing answer. The orthodontic patient without orthodontic malocclusion is not graphically presented in the UpSet plot.

Orthodontic examination showed malocclusions in 99% of children, with crowding (65%), deep bite (54%), and increased overjet (52%) being the most common issues, see Table 1 and the UpSet plot in Figure 2. Maxillary arch constriction (calculated as the difference in the intermolar width corrected for the measurement of the inclination of the lower first molars; see the Supplementary Methods for the full calculation) was present in 47% of patients. Cephalometric analysis revealed 34% of participants to be skeletal class II, 6% skeletal class III, and 60% skeletal class I. The tongue range of motion was physiological in 79% of patients. Oral breathing preference, both daytime and nighttime, was detected in 47% of children. ENT examinations, performed in a subset of 48 children, showed that most examined children had a physiological size of the nasopharyngeal tonsil, with varying degrees of palatine tonsil size. The distribution of AHI across palatine and nasopharyngeal tonsil grades and across breathing patterns in this subgroup is shown in Supplementary Figure S1, and the full measurement methods are provided in the Supplementary Methods.

### Relationships among AHI and other studied variables

**Figure 3:**
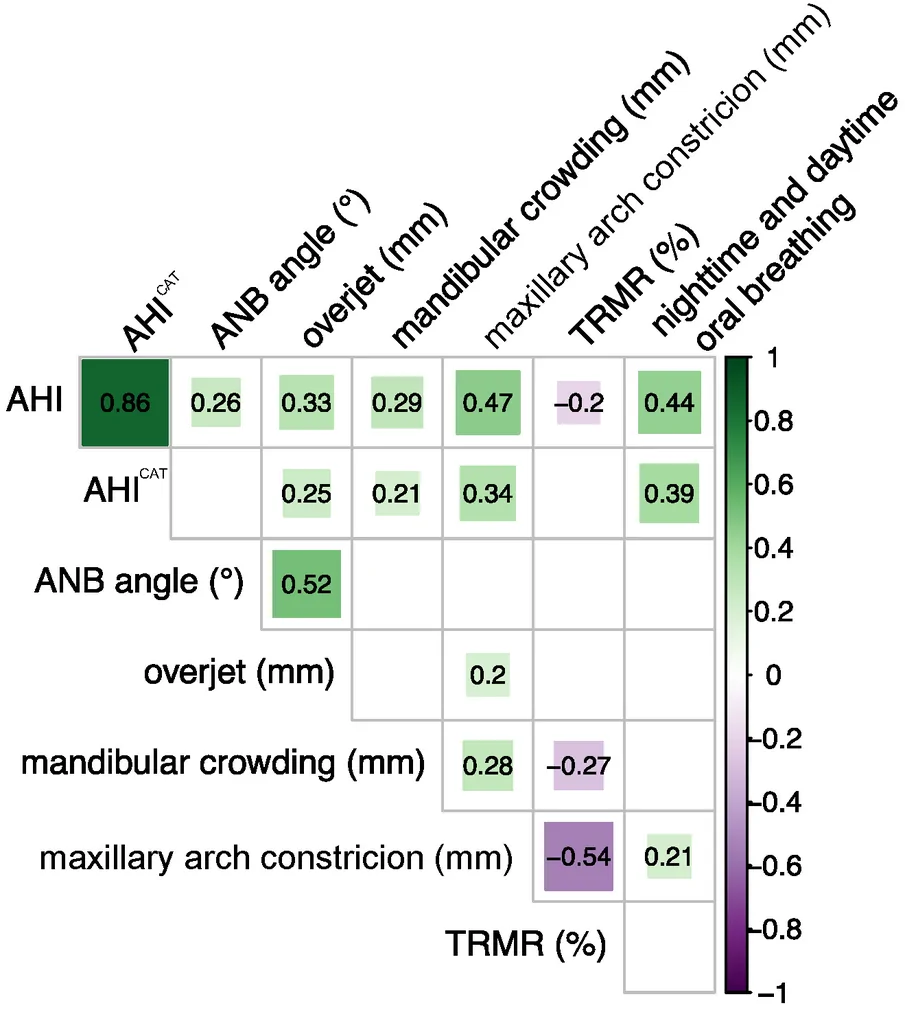
Significant spearman's correlations (r) among continuous apnea-hypopnea index (AHI) and categorized (AHI^CAT^) and examined clinical characteristics. *p < 0.05; **p < 0.01; ***p < 0.001; ANB, angle between points A, nasion, and B; TRMR, tongue range of motion ratio.

Mutual correlations among all studied variables are shown in the Spearman correlation matrix in Supplementary Figure S2. Positive correlations between AHI and the maxillary arch constriction (r = 0.47; *p* < 0.001), mandibular crowding (r = 0.29; *p* < 0.01), overjet (r = 0.33; *p* < 0.001), and the ANB angle (r = 0.26; *p* < 0.01) were found. The tongue range of motion ratio (TRMR) parameter significantly negatively correlated with AHI (r = −0.20; *p* < 0.05). At the same time, we also found positive correlations between the AHI and excessive oral breathing (r = 0.44; *p* < 0.001), see Figure 3.

Throughout this manuscript, the terms mild, moderate, and severe POSA refer to HSAT-based AHI severity categories that estimate the likelihood of POSA, and not to PSG-confirmed diagnoses. In all seven children with AHI ≥ 5, a combination of maxillary arch constriction, crowding, and oral breathing were found. Moreover, all these seven children had at least four co-occurring orthodontic malocclusions, see Figure 2.

**Figure 4:**
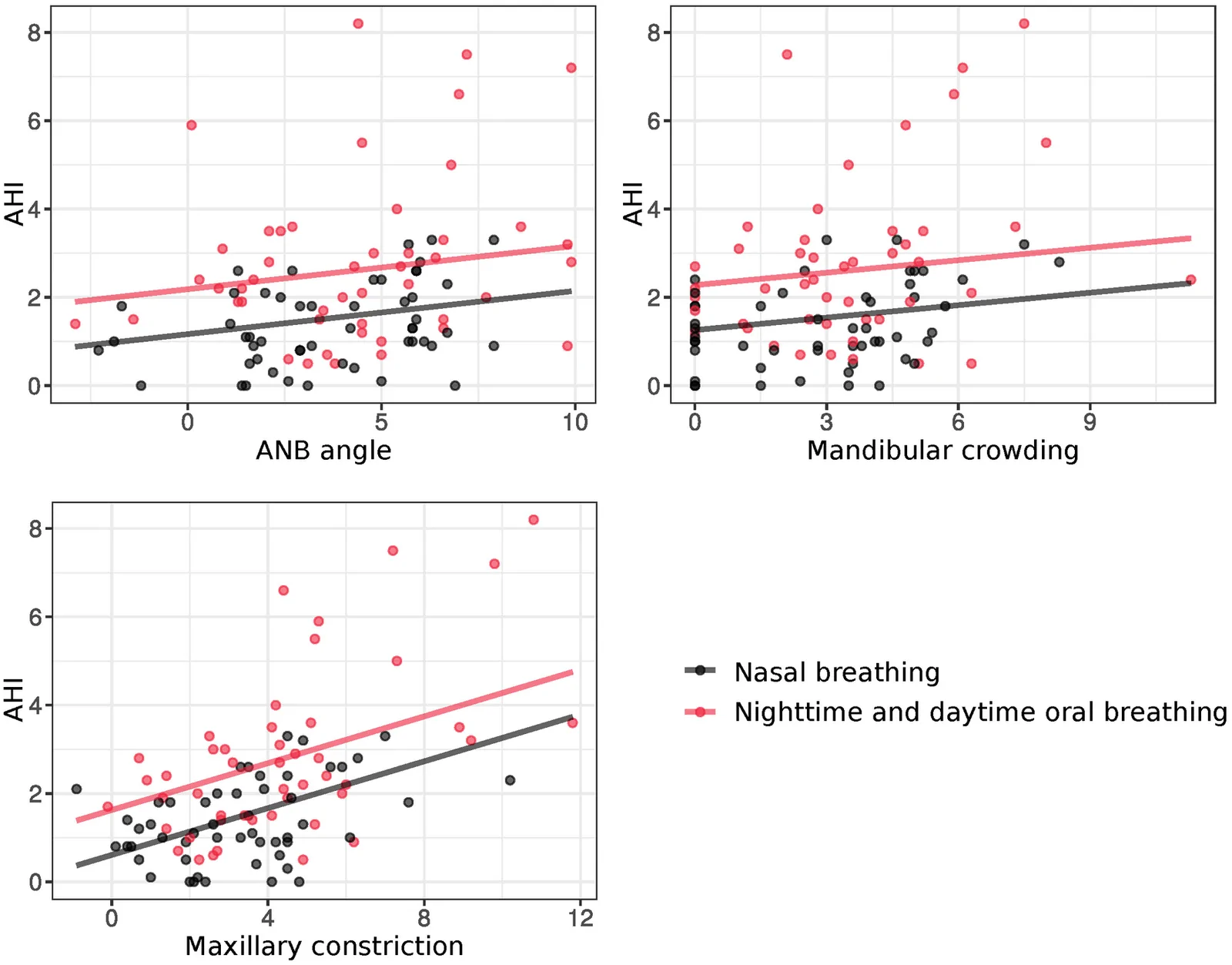
Scatterplots showing the association between the apnea-hypopnea index (AHI) and each of the three continuous predictors, stratified by presence of oral breathing during day and/or night. Points represent individual observations; lines show model-based predictions from the multivariable linear regression, with the remaining covariates held at their median values.

The final linear model excluded all variables but four (increased ANB angle, mandibular crowding, maxillary arch constriction, and nighttime and daytime oral breathing), which, together, explained 77.8% of AHI variance, with the mean error of AHI score prediction of 0.91. Furthermore, diagnostic evaluation of multicollinearity resulted in a maximum adjusted generalized variance inflation factor of 1.85, confirming that collinearity among predictors is negligible. The predictors retained in the final model are presented in Figure 4, and detailed parameter estimates are provided in the Supplementary Table S2.

To evaluate model stability and predictive performance, repeated 5-fold cross-validation was performed. The mean coefficient of determination (R^2^) in the training folds was 0.80, while the mean cross-validated R^2^ was 0.24. The model achieved a mean squared prediction error of 1.63, corresponding to a normalized root mean squared error of 0.79 standard deviations of the observed AHI values. In addition, the model correctly classified participants into the predefined AHI severity categories in 65.5% of validation cases.

The discriminatory performance of the POSA Risk Score (PRS) was further assessed using receiver operating characteristic (ROC) analysis. In the full study cohort, the PRS achieved an area under the ROC curve (AUC) of 0.72 for the identification of children at increased risk of POSA. During repeated 5-fold cross-validation, the mean AUC for the detection of mild POSA (AHI ≥ 1) was 0.71 (SD = 0.12), while the mean AUC for the detection of moderate POSA (AHI ≥ 5) was 0.91 (SD = 0.07), indicating good discriminatory performance, particularly for identifying children at risk of moderate POSA.

The equation below can be used to calculate the POSA RISK SCORE (PRS) as follows:

PRS = − 0.14075

+ 0.09823 × ANB angle [°]

+ 0.09467 × Mandibular crowding [mm]

+ 0.26468 × Maxillary constriction [mm]

+ 1.01392 × Nighttime and daytime oral breathing

where nighttime and daytime oral breathing is a binary variable (1/0).

Based on the resulting PRS value, it is then possible to estimate the risk of POSA in the orthodontic population and the potential need for referring the patient to the sleep specialist. The PRS value < 1 indicates a minimal risk of POSA, values of 1–5 the risk of mild POSA and values > 5 indicate the risk of developing more serious POSA grades (moderate or severe), indicating that the child should be referred to a specialist (note, however, that borderline values need to be interpreted with caution).

## Discussion

The awareness of POSA has increased significantly in recent years, which goes hand in hand with the increasing discussion about the need for interdisciplinary cooperation of specialists on this issue. In many countries, we can see dentists and/or orthodontists constituting important members of the teams involved in the diagnosis and therapy of OSA in adult patients and children (22–24). Even though the awareness of POSA is slowly increasing also in the (country), it still remains often neglected, which leads to underdiagnosis of this problem. Similarly, cooperation in a multidisciplinary team when it comes to the management of POSA also remains relatively rare.

Our first goal was to determine the prevalence of high POSA risk measured in the specific population of children referred for an orthodontic examination in the (country). Our findings contribute to the broader understanding of the risk of POSA in Caucasian children referred for orthodontic evaluation, which implies that the results cannot be directly applied universally to any population. Similar studies in other populations have reported varying POSA prevalence rates, emphasizing the need for region-specific data while also highlighting universal patterns in the associations between craniofacial morphology and POSA risk (25, 26). In the US population, up to 93.3% of children referred for PSG had craniofacial features increasing the risk of POSA (27); in the Malaysian population, authors of a recent publication alerted to the fact that 30% of children seeking orthodontic care were at higher risk of POSA (25).

In our study group, an increased risk of mild/moderate POSA was detected in 68% of children; note, however, that our paper does not speak of confirmed diagnoses of POSA, but of increased risk of POSA. This might appear very high; it is, however, explicable by the character of our study group with an increased prevalence of craniofacial features risky for upper airway narrowing and collapse. In addition, there was an attrition of 33.8% (51 of the 151 initially eligible children). It is possible that parents of children experiencing some symptoms of POSA were more motivated to undergo and complete the home sleep testing, leading to a self-selection (volunteer) effect at the sleep-monitoring stage, at which most of the attrition occurred. Children who completed HSAT may, therefore, have differed systematically from non-completers in symptom burden, parental concern, and motivation, which could have increased the apparent prevalence of POSA risk in the analyzed group and should be considered when interpreting our results.

According to the literature, several orthodontic malocclusions present a risk for airway constriction and POSA, including micrognathia, constriction of the nasomaxillary complex, arched and narrowed hard palate, retrognathic position of the mandible, or its posteriorotation (28–31). Fagundes et al. conducted a systematic review and meta-analysis identifying craniofacial features associated with pediatric obstructive sleep apnea (POSA) (32). They found that children with POSA often exhibit reduced maxillary transverse dimensions, increased mandibular plane angle, and retrognathic mandibles. Our findings align with previous studies indicating associations between craniofacial morphology and increased POSA risk; it is crucial to recognize that the overall quality of evidence supporting this link remains limited. The existing studies often demonstrate low to moderate certainty, emphasizing the need for larger, well-designed research to establish more robust conclusions (32, 33). Additionally, another study by Fagundes et al. investigated whether craniofacial phenotypes could be identified in POSA patients; its results did not identify any distinct phenotypes based solely on soft tissue facial features or craniofacial abnormalities. This indicates that craniofacial characteristics alone may not be sufficient to generalize findings across all POSA cases (34). However, recent systematic reviews concluded that the craniofacial differences observed between children with POSA and healthy controls are often minimal and may lack clinical significance (32–35), In contrast, our study found strong associations between the craniofacial morphology – particularly maxillary arch constriction, increased ANB angle, and mandibular crowding – and AHI. We believe this discrepancy may be explained by the nature of our study population. Unlike the meta-analyses comprising relatively heterogeneous groups of patients, our study was performed in a homogeneous group of children with uniform genetic background who were referred for orthodontic assessment, in which craniofacial abnormalities are overrepresented. This, together with the aforementioned volunteer effect, might have led to the enrichment of the final study group with children with elevated POSA risk. This highlights the value of orthodontic populations as a high-yield group for early POSA screening, while also emphasizing the need for external validation in more diverse cohorts.

In our study, we have investigated a range of orthodontic malocclusions, oral breathing, and basic characteristics to thoroughly analyze the factors potentially influencing the etiology of POSA. Our linear regression model included four clinically relevant characteristics (maxillary arch constriction, mandibular crowding, increased ANB angle, and nighttime and daytime oral breathing) that most significantly contribute to the risk of POSA. Although the model showed relatively low mean validation R^2^ of 0.24 (when splitting the data to training/validation 80%/20% and performing 1000x repeated cross-validation), the AUC for identification of children at risk of POSA (0.72) and, particularly, at risk of moderate POSA (AUC = 0.91) remained relatively high.

While oral breathing preference showed the strongest individual correlation with AHI, the effect sizes of the remaining features included in the model were lower. Despite this fact, their inclusion in the model significantly improved its predictive strength. This supports the concept that POSA risk arises from the additive influence of multiple, partially independent, risk factors, each contributing with a different weight. Consequently, focusing solely on strong individual predictors (and omitting weak predictors with significant association) might lead to underestimation of the clinical relevance of subtler morphological features.

It was also shown that greater maxillary arch constriction was associated with higher AHI. A narrow upper jaw is also often mentioned as a significant risk factor for airway obstruction, because it plays a role in the reduction of the volume of the nasal cavity, which may further contribute to nocturnal hypopnea or apnea and to an increase in AHI score (36). This is also in agreement with studies that identified a narrow nasomaxillary complex, arched, and/or narrowed hard palate as frequent findings in children with POSA. These studies also described a positive effect, up to a complete disappearance of POSA symptoms, after rapid maxillary expansion (12, 27, 37). In our patients, we found a positive correlation between mandibular crowding and higher AHI, which was also found in the study by Pirilä-Parkkinen et al. (38). The correlation between maxillary crowding and the severity of POSA was not statistically significant in our study. However, our results suggest that although the maxillary crowding alone may not significantly influence AHI, the overall shape and constriction of the maxillary arch do.

As some recent studies suggested that the 2 mm difference between the facial axis of the lower first molars and the WALA ridge reported by Andrews may be arbitrary, it could be objected that the WALA ridge measurement used in our study overestimates the palatal arch constriction (20, 21). To account for this, we also performed an additional analysis of correlation between the maxillary intermolar width alone and AHI and revealed a significant negative correlation between these two factors (r = −0.41; *p* < 0.001) This further corroborates the finding that the maxillary arch constriction is a very important predictor of AHI.

We have further found a negative correlation between the TRMR parameter and maxillary arch constriction. Our observations are also supported by studies that describe the association between the non-physiological lower position of the tongue, often caused by a short sublingual frenulum, and the maxillary constriction (39). The TRMR was negatively correlated not only with maxillary arch constriction but also with AHI itself. As such, we can propose it as another risk factor for the development of POSA.

Some studies have mentioned the posteriorotation of the mandible, particularly an increase in the sellanasion/mandibular (SN-ML) angle as another parameter associated with POSA (30, 31). In our study, however, we did not observe this relationship. It needs to be pointed out that the observed association between craniofacial morphology and airway obstruction may be bidirectional. While specific craniofacial features may predispose a child to POSA, the presence of upper airway obstruction due to enlarged adenoids and tonsils can, in turn, influence craniofacial growth and development. This bidirectionality, together with the fact that adenoid and tonsil size was available for fewer than half of our participants (48 of 100), means that part of the association attributed to craniofacial morphology could in reality reflect unmeasured lymphoid-tissue obstruction (residual confounding), which should be considered when interpreting the craniofacial predictors.

Several studies have shown a link between chronic oral breathing and impaired orofacial development (12). This leads to the emergence of characteristics leading to airway narrowing and difficult nasal breathing, which, in turn, further strengthens oral breathing preference (40). Our premise that oral breathing, especially this respiratory pathology in both day and night, is a risk factor for POSA (i.e., that it is associated with higher AHI), was confirmed in our study. A positive correlation between oral breathing and maxillary arch constriction was also observed, which is in line with studies that indicate oral breathing as well as craniofacial dysmorphisms to be both risk factors and symptoms of POSA (40, 41).

### Limitations and strengths

As mentioned in the methodological part of our study, we are aware that the definite POSA diagnosis can be only performed using PSG and that our test does not provide this definite diagnosis. Still, the used HSAT method (respiratory polygraphy) has been previously recommended as a suitable diagnostic method, especially in cases of the unavailability of PSG (42, 43). Our meticulous manual verification of all sleep records, removing obvious non-sleep periods, allowed us to approximate the total sleep time (TST); its use instead of the total recorded time (TRT) allowed us to consider the acquired parameter to be AHI. However, this measurement still can be considered a limitation of our study; hence, we use the terminology of “higher risk of POSA” rather than the definite POSA diagnosis.

Although all participants were referred for ENT evaluation, only a subset underwent a complete ENT examination due to practical limitations during the COVID-19 pandemic. In effect, this information was available only for 48/100 patients. Despite acquiring surgical history from all parents, we observed some inconsistencies between parent-reported history and the clinical findings. To remove this possible source of inaccuracy, we decided to exclude the unverified parental reports from the analysis. At the same time, however, this meant that we were unable to include values (particularly the adenoid/tonsil size) into the model, which could, therefore, suffer from residual confounding and can be only interpreted as preliminary. Including ENT values can further improve its quality and is highly recommended for future studies. For the evaluation of the oral breathing preference during the day, we used the visual assessment of lip seal, clinical examination of nasal movements, and the child's medical history acquired from the parents as often used for diagnosing oral breathing (44); however, nighttime oral breathing parameter was evaluated only on the basis of parents’ cooperation – they were asked to watch their child sleeping for several nights, evaluate the breathing pattern, and report it to us. A further limitation is that, although age, sex, and BMI z-score were collected, pubertal status and a structured assessment of respiratory/atopic allergies were not recorded; both are recognized modifiers of POSA risk, and their absence means we cannot fully exclude residual confounding from these factors. Lastly, no formal sample size calculation was performed prior to the study. This is, however, completely in line with the fact that this was an exploratory observational study designed rather to generate data that can be used for sample size calculations in future, larger, confirmatory studies than for the generation of definite results. External validation in broader and more diverse cohorts is necessary before the model can be recommended for clinical application to other populations.

On the other hand, the strong points of the study include (a) a relatively large patient population, (b) the homogeneity of the population and examination of a wide range of parameters, and (c) the construction of the linear model that identified four parameters (maxillary arch constriction, mandibular crowding, oral breathing, and increased ANB angle) to be most strongly associated with POSA. The model based on these parameters has the potential to become a useful tool for the prediction of the risk of POSA, which can help in the preselection of orthodontic patients. Our findings are based on a relatively homogeneous orthodontic population in (country), which may limit the generalizability of the results.

## Conclusions

In our population, the evidence of increased POSA risk based on a respiratory polygraphy was detected in 68% of children referred for orthodontic examination. A significant association between AHI and several craniofacial characteristics (most importantly the maxillary arch constriction, mandibular crowding, oral breathing, and increased ANB angle) was found. The results of the final linear regression model based on these four variables successfully explained 77.8% of the variance in AHI scores, very well predicting the AHI (with a mean error of prediction being 0.9 AHI points).

## Data Availability

The original contributions presented in the study are included in the article/Supplementary Material, further inquiries can be directed to the corresponding author.
