# Supplementary material for: Risk of pediatric obstructive sleep apnea in preadolescent children with orthodontic anomalies: a diagnostic study
Source: Front Oral Health. 2026 Aug 25;7:1868634. doi: 10.3389/froh.2026.1868634 (PMC13549837; doi:10.3389/froh.2026.1868634)
Supplement: Supplementary file 1 [file Supplementaryfile1.pdf]

Risk of pediatric obstructive sleep apnea in preadolescent children with orthodontic anomalies: A diagnostic study – Supporting information

Supplementary Methods

Clinical examinations

The BMI categorization was performed based on the z-score; children were classified as "obese" for z-scores 2 to 3, as "overweight" for z-scores 1 to 2, "normal weight" for z-scores -2 to 1, and "underweight" for z-scores -3 to -2 [1]. All patients enrolled in the study underwent a home sleep apnea examination, a complex orthodontic examination including two X-ray images, and were also referred to a specialist for a complex ear, nose, and throat (ENT) examination.

Home sleep apnea test – respiratory polygraphy

The all-night sleep examination was performed in all patients using a home sleep apnea test – (polygraphy, HSAT). The patients' parents were instructed on the handling of the sleep monitor – the Alice NightOne device by Philips Respironics (Respironics, Inc., Murrysville, PA 15668, USA). This device, consisting of several sensors recommended by the American Academy of Sleep Medicine, keeps monitoring the position of the body, airflow pressure, snoring, respiratory effort, the level of oxygen saturation (SpO_2), and the pulse [2]. Apnea was defined as a drop in the signal excursion by ≥90% of the pre-event baseline for ≥10 seconds or two breath cycles, and hypopnea was defined as a drop by ≥30 % of the pre-event baseline using nasal pressure, followed by a ≥3% oxygen desaturation [3]. The risk of POSA is determined based on the apnea-hypopnea index (AHI), i.e., the number of apneas and hypopneas per hour of sleep. In our study, we have used the scoring recommended for children under 12 years, which is AHI 0–1 (physiological finding), AHI 1–5 (mild POSA), AHI 5–10 (moderate POSA), or AHI > 10 (severe POSA) [4]. In HSAT, results are usually expressed as the respiratory event index (REI) instead of AHI as HSAT typically uses the total recorded time (TRT) instead of the total sleep time (TST). This is because HSAT cannot reliably distinguish between sleep and awake phases without EEG monitoring. However, the use of TRT and REI can lead to an underestimation of the severity of sleep apnea [5]. In our study, all records were manually scored and analyzed in Sleepware G3, version 3.9.6.0 program (Respironics, Inc., Murrysville, PA 15668, USA) by a doctor trained in the field of sleep medicine. During this process, the doctor also removed obvious non-sleep periods from the records identified based on breathing patterns and the child's movements. This approach allowed us to approximate and use the TST, thus acquiring a value that can be considered rather AHI than REI and improving the interpretation and categorization of risk for POSA in our patients. Selected records were also externally consulted with the Cardiovascular Sleep Laboratory, St. Anne's University Hospital (Brno, Czech Republic). A recording was considered evaluable only when at least 5 hours of analyzable,

artifact-free signal (airflow and oxygen saturation) were available; recordings below this threshold were classified as unsuitable for evaluation.

Orthodontic examination

The initial orthodontic examination consisted of extraoral and intraoral examination, during which the patient's overall habitus, the presence of all orthodontic anomalies, *facies adenoidea*, and oral breathing were recorded. This complex examination included both intraoral and extraoral photographs, dental impressions, and two X-rays – orthopantomogram (OPG) and cephalogram. After a detailed analysis of all the obtained data, including the measurements of maxillary constriction, the length of the sublingual frenulum, tongue mobility, and the analysis of both X-rays (all described in detail below), the orthodontic diagnosis was established, and the therapeutic plan was prepared.

Orthodontic anomalies were evaluated in several key areas, such as the: a) number of teeth (supernumerary tooth, tooth agenesis), b) position of teeth (tooth retention, single tooth crossbite, posterior crossbite, anterior crossbite, crowding), c) relationship of dental arches in different planes (overjet, overbite); with the emphasis on anomalies that have been previously associated with possible higher risk of POSA. Each anomaly was recorded either categorically (yes/no) or as a continuous parameter (in millimeters or degrees). In some cases, anomalies were documented using both methods. For example: a) overjet was considered an anomaly if it exceeded 3 mm, [6] b) overbite was classified as a deep bite if it was more than 3 mm or as an open bite if it was less than 0 mm, [6] c) crowding was noted if there was more than a 3 mm discrepancy in the maxillary or mandibular arch, [6] d) maxillary arch constriction was considered as anomaly in our study if it exceeded 4 mm. While these categorizations helped illustrate the important findings, the exact values were used wherever possible for all our analyses and statistical calculations.

Maxillary constriction is usually clinically determined based on the presence of uni- or bilateral crossbite. Even though we have examined this factor in our patients, the maxillary arch may be constricted even in the absence of the crossbite. In the presented study, we have chosen to use the measurement of intermolar width, considering it is a well-known and often used type of measurement. As a second step, we have also combined it with the evaluation of the WALA ridge reference (the most lateral and inferior aspect of the alveolar bone on the mandible, at the level of the mucogingival junction, initially described by Andrews and Andrews) to consider also the discrepancy that could be masked by the inclination of the lower molars [7]. By integrating these measurements, we achieved a detailed understanding of the maxillary arch constriction, considering both absolute measurements and positional inclinations.

First, all plaster models were scanned using an iTero Element 5D Plus intraoral scanner (Align Technology Inc., San Jose, CA, USA). We determined and recorded the distance from the WALA ridge to the facial axis (FA) point separately for the right and left inferior first molars. If the distance was greater or less than the specified 2 mm, we calculated the difference. This difference indicates whether

the lower first molars are in their ideal (decompensated) position or if they are inclined. A significant deviation from 2 mm suggests that the molars are not in their optimal position, as described by Andrews and Andrews [7].

In the second step, we measured the intermolar width, the distance between the fissures of the lower first molars, and the distance between the mesiopalatine cusps of the upper first molars. These measurements were then compared. Ideally, when the widths of the dental arches correspond, the two measurements should be the same. Any discrepancies between these values indicate a mismatch in the widths of the dental arches.

In addition to the comparison in the second step, we accounted for the measured difference between WALA and FA from the first step. This comprehensive measurement allowed us to account for not only the narrowing of the maxilla relative to the mandible but also any discrepancy in the width of the dental arches that might be masked by the inclination of the lower molars.

The examination of the sublingual frenulum should be included in the initial orthodontic examination as its shortening could have several consequences on the orofacial growth and, moreover, could represent a risk factor for sleep-disordered breathing [8]. In our study, the examination consists of a functional examination of the tongue movement, which may be impaired, especially in the case of shortening of the frenulum. For this purpose, we used the measurement described by Marchesan [9].

The examination began with visual and tactile inspection, followed by an assessment of the functional movements of the tongue, which can be particularly affected in cases of frenulum shortening. Frenulum shortening was measured using a stainless steel tongue-tie assessment tool. Two measurements were taken: a) the interincisal distance between the upper and lower central incisors at maximum mouth opening (maximal interincisal opening, MIO), b) the interincisal distance when the patient was asked to lift the tongue tip to the upper central incisors or the incisive papilla and open the mouth as wide as possible again (interincisal mouth opening with tongue tip to maxillary incisive papillae – MOTTIP). Subsequently, the tongue range of motion ratio (TRMR) was calculated. This value is determined as the ratio between MOTTIP and MIO, expressed as a percentage. According to the study by Marchesan et al.[9], the interpretation of results is as follows:

TRMR > 60% indicates a physiological frenulum with normal tongue mobility.

TRMR < 50% indicates an abnormal sublingual frenulum with restricted tongue mobility.

TRMR = 51-59% yields an unclear result.

All patients included in the study had two X-ray images taken in a standardized way (CS 9300, Carestream Dental, USA). In the analysis of the X-rays, we have focused on both dental and skeletal anomalies, on the agenesis of teeth, and parameters that have already been shown to be associated with POSA, such as the SNA angle, SNB angle, ANB angle, SN-ML angle, and hyoid bone position. Cephalometric images were analyzed at the Orthodontic Department of the Clinic of Stomatology, St. Anne's University Hospital (Brno, Czech Republic), using the Ortik cephalometric software (Orthosoft s.r.o., Czech Republic). Physiological ranges of individual parameters were adopted from the paper by

Jarabak and Fizzell [10] as follows: skeletal class II, i.e., the ANB angle > 5; skeletal class III. with ANB angle < -1; and skeletal class I. with ANB angle [-1, 5]. Based on the SN-ML measurements, we classified the patients into categories of a) neutral mandibular growth [28°, 36°], b) hyperdivergent skeletal pattern (SN-ML > 36°), and c) hypodivergent skeletal pattern (SN-ML < 28°).

Measurement error

The measurement error was determined as part of the analysis of cephalometric images and the measurement of maxillary constriction. For this purpose, all 100 images and 100 patient model scans were analyzed three times in succession, each with a 14-day interval. To eliminate random or systematic error, a double check of the cephalometric point placement was conducted. Fourteen days after the first measurement, the position of the points was rechecked for all cephalometric analyses, and then another check was performed 14 days after the previous measurement.

The obtained data from the scanned models and cephalometric analyses were compared. The measurement error was checked using the Dahlberg formula (D). Since the calculation of the error using Dahlberg's method does not take into account the size of the measured values, the relative Dahlberg error (RDE) was also used to compare the accuracy of the measurements for individual parameters. This is obtained by dividing the Dahlberg error by the average of the corresponding measured values and multiplying by 100, which gives the result in percentage. The degree of absolute agreement between the first and control measurements was verified by calculating the ICC (Intraclass Correlation Coefficient), and the presence of systematic error was checked using a paired t-test.

Measurement Error in Cephalometric Analyses and Scanned Model Analyses

The relative Dahlberg error ranged from 0.3% to 10.8%. The ICC coefficient values were found to be in the range of 0.991 to 0.999, indicating perfect agreement. The paired t-test did not reveal any systematic error (all *p*-values were greater than 0.05). These values confirm that our measurement was sufficiently accurate – all parameters were measured with sufficient precision and without systematic error. Intraindividual error was low, and the errors were random with no significant trends.

ENT examination

All patients involved in the study were referred for the ENT examination by a pediatric ENT specialist at the Children ENT Ltd., (Brno, Czech Republic). The patients' breathing patterns were examined, with emphasis on their medical history. Subsequently, they underwent an endoscopic examination of the nose and nasopharynx with photo documentation. A flexible rhino-laryngo videoendoscope Olympus (Olympus Czech Group s.r.o., Czech Republic), designed for pediatric patients, was used for endoscopy. This device allowed detailed observation of the nasal passages, the condition of the mucosa, and the possible presence of pathological changes. We recorded and staged the size of palatine tonsils (Grade 0 – 4) and adenoid vegetation (Grade I – III) according to the criteria recommended by the Czech Society for Ear, Nose, and Throat Diseases [11].

Supplementary Figures

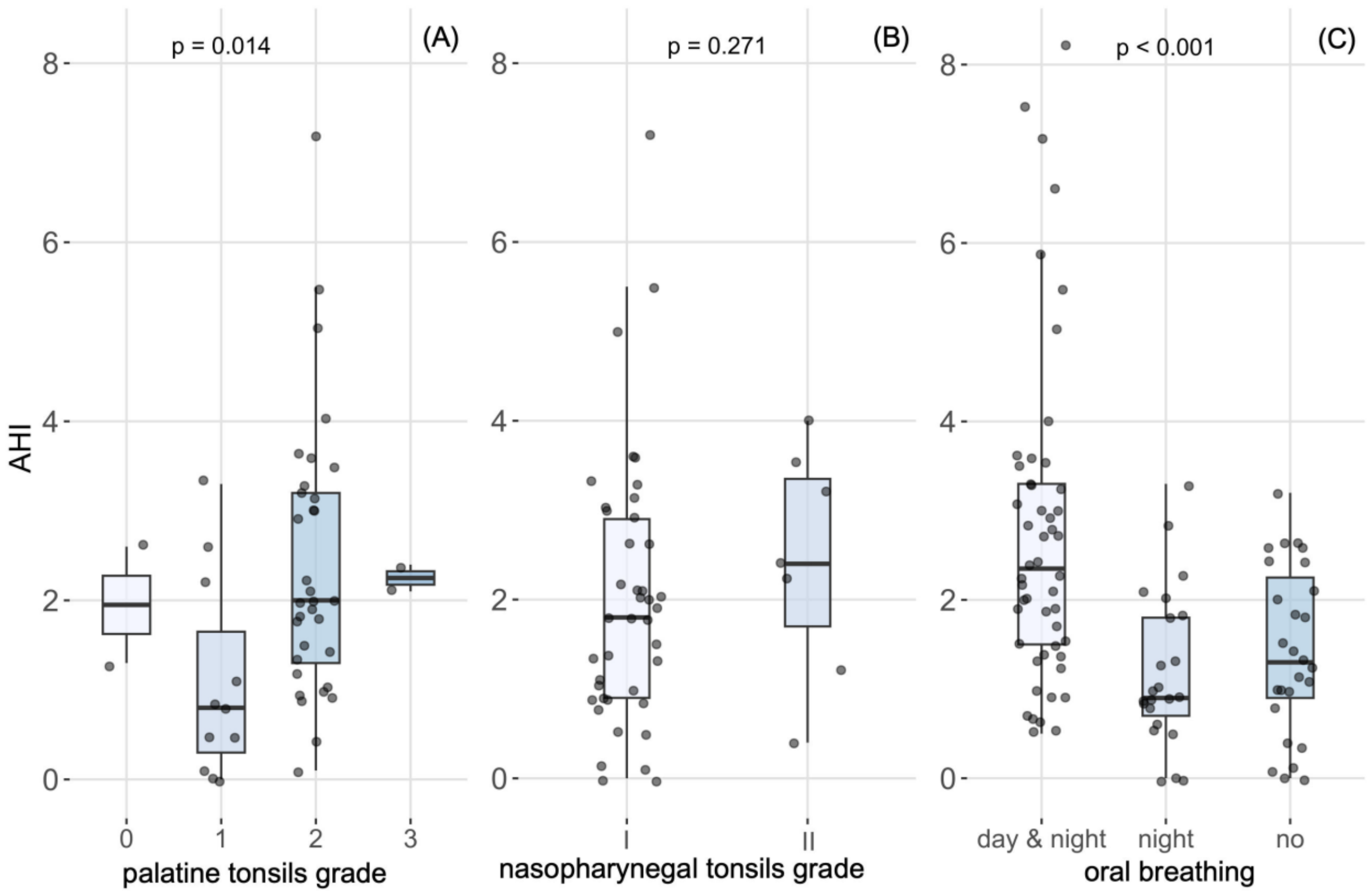

Supplementary Figure S1. Boxplot depicting median apnea-hypopnea index (AHI; black bar) and its IQR (the height of the boxes) in children with different grades of (A) palatine and (B) nasopharyngeal tonsils size, and (C) in children with different breathing patterns. Jittered points depict the AHI of individual children.

Interquartile range, IQR

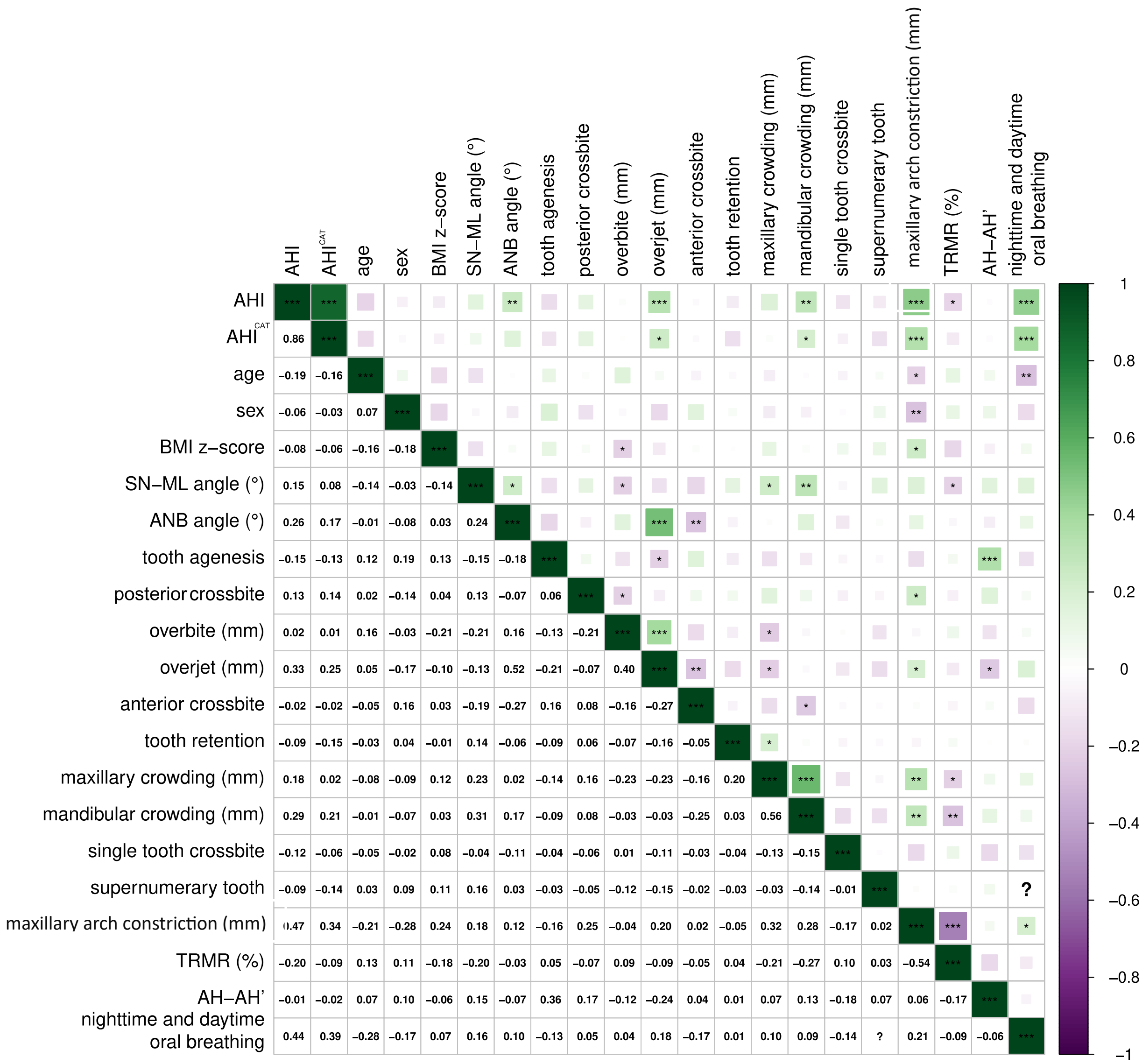

Supplementary Figure S2. Spearman's correlations among all studied variables, including continuous (AHI) and categorized (AHI^{CAT}) apnea-hypopnea index, and examined clinical characteristics

* $p < 0.05$, ** $p < 0.01$, *** $p < 0.001$

AH-AH', the distance of the hyoid bone from the mandibular line; AHI, apnea-hypopnea index; ANB, angle between points A, nasion, and B; BMI, body mass index; SN-ML (sellanasion/mandibular line) angle; TRMR, tongue range of motion ratio

Supplementary Tables

Supplementary Table S1. Measurement errors in cephalometric analyses and scanned model analyses

Parameters	D (°)	RDE (%)	ICC	*p*-value (t-test)
**Intermolar width of the lower first molars**	0.157	6.3	0.999	0.421
**Intermolar width of the upper first molars**	0.124	0.3	0.999	0.366
**FA and WALA 46**	0.052	7.5	0.998	0.348
**FA and WALA 36**	0.056	7.5	0.996	0.277
**Maxillary arch constriction**	0.165	4.3	0.999	0.128
**SN-ML angle**	0.276	0.8	0.999	0.086
**SNA angle**	0.320	0.4	0.997	0.677
**SNB angle**	0.275	0.4	0.998	0.283
**ANB angle**	0.431	10.8	0.991	0.935

FA, facial axis; WALA, the most lateral and inferior aspect of the alveolar bone on the mandible, at the level of the mucogingival junction, initially described by Andrews and Andrews; SN, sella – nasion; ML, mandibular plane; ANB, point A, N – nasion, and point B; SNA, points sella, nasion, and point A; SNB, point sella, nasion, and point B; D, Dahlberg formula; RDE, relative Dahlberg error; ICC, intraclass correlation coefficient

Supplementary Table S2. Regression coefficients and corresponding statistics used in the construction of the linear regression model for clinically relevant craniofacial features correlating with AHI in pediatric orthodontic patients

Coefficients	Estimate	Std. error	95% CI lower	95% CI upper	t-value	*p*-value
**Increased ANB angle (°)**	0.098	0.046	0.008	0.188	2.116	0.037
**Mandibular crowding (mm)**	0.095	0.059	-0.021	0.211	1.613	0.110
**Maxillary arch constriction (mm)**	0.265	0.056	0.155	0.374	4.706	< 0.001
**Nighttime and daytime oral breathing (yes)**	0.873	0.352	0.183	1.563	2.478	0.015
**Nighttime and daytime oral breathing (no)**	-0.141	0.297	-0.723	0.441	-0.474	0.636

AHI, apnea-hypopnea index; ANB, angle between points A, nasion, and B; CI, confidence interval

Supplementary References

1. Dean A, Arner T, Sunki G, et al. Epi Info™, a database and statistics program for public health professionals. CDC, Atlanta, GA, USA. 2011
2. Thornton AT, Singh P, Ruehland WR, Rochford PD. AASM criteria for scoring respiratory events: interaction between apnea sensor and hypopnea definition. Sleep. 2012;35(3):425-432.
3. Grigg-Damberger MM. The AASM Scoring Manual four years later. J Clin Sleep Med. 2012;8(3):323-332.
4. Rubinstein BJ, Baldassari CM. An update on the management of pediatric obstructive sleep apnea. Curr Treat Options Pediatr. 2015;1:211-223.
5. Pitkänen M, Nath RK, Korkalainen H, et al. Respiratory event index underestimates severity of sleep apnea compared to apnea-hypopnea index. Sleep Adv. 2023;5(1):zpad054.
6. Proffit WR, Fields H, Larson B, Sarver DM. Contemporary Orthodontics-E-Book: Contemporary Orthodontics-E-Book. Elsevier Health Sciences; 2018.
7. Andrews LF. Andrews WA. The six elements of orofacial harmony. Andrews J.Orthodont. Orofac. Harmony 2000;1:13-22
8. Guilleminault C, Huseni S, Lo L. A frequent phenotype for paediatric sleep apnoea: short lingual frenulum. ERJ Open Research. 2016;2(3)
9. Marchesan IQ. Lingual frenulum: quantitative evaluation proposal. Int J Orofacial Myology. 2005;31:39-48.
10. Jarabak JR, Fizzell JA. Technique and treatment with light-wire edgewise appliances. vol 1. CV Mosby Company; 1972.
11. Ondrová M, Plzák J, Lánský M, Hrdlička A. Doporučený postup u dětských pacientů s poruchami dýchání ve spánku. Otolaryng. a Foniat. 2016;4:247-248.
